# HIV Prevalence and Correlations in Prisons in Different Regions of the World: A Review Article

**DOI:** 10.2174/1874613601812010081

**Published:** 2018-08-31

**Authors:** Raheleh Golrokhi, Behnam Farhoudi, Leila Taj, Fatemeh Golsoorat Pahlaviani, Elham Mazaheri-Tehrani, Andrea Cossarizza, SeyedAhmad SeyedAlinaghi, Minoo Mohraz, Fabrício Azevedo Voltarelli

**Affiliations:** 1 Iranian Research Center for HIV/AIDS, Iranian Institute for Reduction of High Risk Behaviors, Tehran University of Medical Sciences, Tehran, Iran; 2 Clinical Research Development Center, Amir-Almomenin Hospital, Tehran Medical Sciences Branch, Islamic Azad University, Tehran, Iran; 3 University of Modena and Reggio Emilia School of Medicine, Modena, Italy; 4 Federal University of Mato Grosso, Graduation Program in Health Sciences, Faculty of Medicine, Cuiabá, Brazil

**Keywords:** HIV prevalence, HIV risk factors, Prison, Injecting drug use, Sexually transmitted diseases, Correlations

## Abstract

The prevalence of HIV is substantially higher among prisoners than the general population, while the incidence varies considerably in different regions around the world. If we consider Sub-Saharan Africa as one region with the highest prevalence of HIV, data on African prisoners would be limited. Despite the low prevalence of HIV in the Middle East and North Africa, its incidence is rising in these regions with a few exceptions; there are insufficient data on HIV prevalence in prisons. A similar situation is present in both Pacific and Central Asia as well as in Eastern Europe. A high rate of infection is mainly observed among prisoners in Western and Central parts of Europe, since the data from these are more available than other parts. Nowadays, the sexual transmission mode and tattooing are important ways in HIV risks among prisoners after injecting drug use as the most common route of HIV transmission in all regions. However, it is difficult to compare and analyze the prevalence of HIV among prisoners in different regions regarding the limited data and different methods which they used in collecting data. Eventually, it can certainly be said that prisons are one of the high-risk places for HIV transmission; on the other hand, can be a suitable place for implementing HIV case-finding, linkage to treatment and harm reduction programs.

## 1 INTRODUCTION

The world population of prisoners in 2014 was estimated about 10.2 million and incidence of HIV infection among prisoners was much higher than the general population [1-3]. For example, the rate of diagnosed HIV infection among federal inmates in the United States prisons was more than five times greater than this rate among people who were not incarcerated (2.0%). It is probably because of the low socioeconomic level of prisoners, illiteracy, history of drug injection, high unsafe sexual behavior and tattooing [4]. The large number of HIV-infected inmates makes prisons potential places for the widespread of HIV [5, 6]. This risk increases the necessity of identifying inmates living with having a risk of HIV infection, providing HIV testing and appropriate services for HIV treatment and prevention [7].

The impacts of HIV intra-prison transmission are not restricted to those aforementioned incarcerated: considering the average lengths of jail stay (< 3 months), the risk for continued HIV transmission stretches to the general community and re-entry programs of the ex-offender. Thus, interventions for HIV prevention that are addressed in prisons affect not only the prisoners, but also the larger communities outside the prisons [8].

In most countries, HIV prevalence rates in prison are several times higher than in the community outside the prisons, and this is closely related to the rate of HIV infection among people who inject drugs in the community and the proportion of prisoners convicted for drug-related offences [9-17]. Incarceration provides an opportunity to implement HIV prevention programs for prisoners [18].

By 2015, the National HIV/AIDS Strategy for the US (updated to 2020) outlines four goals, including: i) reducing HIV incidence; ii) increasing access to care and improving health outcomes for HIV infected people; iii) reducing HIV-related disparities and health inequalities; and iv) achieving a more coordinated national response to the HIV epidemic [19]. To achieve these goals, it is important to be aware of HIV prevalence and its related risk factors in prisons. Accordingly, the aim of this review was to produce a coherent summary of HIV prevalence and the correlates among the prisoners.

## 2 SEARCH STRATEGY

We searched some important databases such as PubMed and Google Scholar between January 1, 1991 to April 30, 2018 by using some keywords and phrases such as: “HIV prevalence in prison”, “HIV risk factors + prison”, “HIV transmission + prison”, “blood transmitted diseases in prison”, “sexually transmitted diseases in prison”, “HIV prevalence in Middle East and North Africa (MENA)”, “HIV prevalence in Asia and Pacific”. We also analyzed the Joint United Nations Programme on HIV/AIDS (UNAIDS) documents to find more information related to the prisoners.

## 3 HIV PREVALENCE IN PRISONS WORLDWIDE

### 3.1 Sub-Saharan Africa

HIV prevalence among prisoners in this region varies from 2.3% to 34.9% among different regions [20]. Southern Africa is the most affected country, and it is widely considered as the 'epicenter' of the HIV epidemic worldwide [21]. Swaziland has the highest HIV prevalence in the world (27.4%), while South Africa has the largest epidemic in the world with 5.9 million people living with HIV [21]. Comparatively, HIV prevalence among prisoners in Western Africa is ranged from 2.3% to 10.8% and in Eastern Africa is varied from 4.2% to 23% [20]. In a study in Zambia among 1,596 prisoners, 421 (27%) were seropositive [22]. A study in Ghana among 281 inmates participating to an HIV screening program showed a rate of infection of 19.2% [23]. United Nations Office on Drugs and Crime (UNODC) research on 459 prisoners in 34 Uganda prisons found that the prevalence of HIV was 11% among those prisoners [24]. Also, in a research in Burkina Faso 5% of 300 prisoners were affected by HIV infection [14].

Unfortunately, there are limited data on HIV in prisoners living in other countries from this part of the world, and those available are not appropriately accurate or not enough to provide a representation of the current situation in this area.

### 3.2 Middle East and North Africa

Rates of HIV infection in the MENA are among the lowest in the world, even in this region HIV is still rising [25]. Although prisoners are considered a high-risk group for HIV, prevention programs involving this group are limited. Iran has had little advancement in addressing HIV transmission in prison settings; by using harm reduction programs that include HIV testing and counseling, providing information, education and opioid substitution therapy. In a study performed in Iran, a clinical guideline was implemented for HIV care and treatment services in prisons, which was successful in HIV case finding and provision of its care and treatment. The guideline has been used in 16 prisons of 13 provinces [26]. Today, an increasing number of MENA countries such as Egypt, Lebanon, Morocco, and Tunisia are also trying to address HIV transmission among their prisoners, but there is no accurate and reliable data to show HIV prevalence in prisons of this region.

In a nationwide bio-behavioral study in Iran, conducted in 27 prisons, among 5,530 prisoners recruited the prevalence of HIV infection was 2.1% (95% CI: 1.2 to 3.6) [27]. In a study of 2012-2013 using the same method, the prevalence of HIV infection among Iranian prisoners was %1.4 (95% CI: 0.6-2.2). The study suggested no significant change comparing to previous study. The HIV prevalence for those who had a history of drug injection was 5.42% (95% CI: 2.09-8.76) [28]. In three studies conducted in Iran to evaluate the HIV prevalence among drug users, results were 15.1% among 252 prisoners in Bandar Abbas and Roodan [29], 5.8% among 121 prisoners in Gorgan [30] and 6.4% among 970 prisoners in Isfahan [31]. Another study has been performed in Iran among 459 IDU males arrested by the police. In this group, the prevalence rate of 24.4% has been reported [32].

### 3.3 Asia and Pacific

After Sub-Saharan Africa, the regions with the largest number of people living with HIV are Asia and the Pacific. At the end of 2015, there were an estimated 5.1 million people living with HIV across this region. There has been a slow progress in reducing number of new HIV infected cases in recent years, and also there are rising epidemics in some countries [33].

In two studies in Pakistan, the prevalence rate among prisoners has been estimated less than 1% [34, 35] while, in a review study in India, it has estimated about 1.7% [36]. Following, a study performed in Australia reported a prevalence of 0.47%, approximately [11]. The incidence of infection in these inmates was 4.2 per 100 person/year among all inmates, and 11.1 per 100 person/year among the IDU rests on a study in Bangkok [37].

### 3.4 Eastern Europe and Central Asia

In comparison to Western and Central Europe, HIV epidemic in Eastern Europe and Central Asia continually grows, particularly in Russia, Ukraine and Uzbekistan. Actually, about 85% of people living with HIV in this part of the globe are living in Russia and Ukraine [38]. In 2010, it was reported that 55,000 of Russian inmates out of 846,000 were thought to be living with HIV. Moreover, four studies from Estonia showed the prevalence of HIV among prisoners between 8.8% and 23.9% [39]. HIV among prisoners is a growing concern in central Asia, especially in Tajikistan and Kyrgyzstan that HIV prevalence among inmates was reported 8% [40].

### 3.5 Western and Central Europe and North America

The United States of America (USA) accounts for the majority of people living with HIV in these regions (56%). In Western Europe, four countries including France (8%), Spain (6%), United Kingdom (5%) and Italy (5%) [21] account for a further quarter of this number. In 2014, HIV transmission due to injecting drug use accounted for 3% of all new HIV infections in Western Europe and 5% in Central Europe [41]. In a study in Greece, the prevalence rate in 544 drug users imprisoned for drug related offences was 0.19% [9].

In a study in Scotland, 162 inmates were tested, 7% were seropositive [42]. The prevalence rate in Ireland has been estimated 2% (24 out of 1205 prisoners) [6] and 6.1% in the USA among 975 newly sentenced male prisoners [43]. In a study in Rhode Island among 4,269 male inmates, 1.2% were HIV positive [8].

In a study in British Columbia, Canada, 2482 adult inmates who admitted to provincial prisons were tested for prevalence of HIV antibodies and 28 individuals (1.1%) were positive [44]. In another province of Canada, Quebec, HIV prevalence was 2% (11 out of 499) in men and 8% (9 out of 119) in women [18]. Additional study including 1616 inmates was conducted in Quebec showed similar results, which was 2.3% among the male and 8.8% among the female participants [45]. Among 1877 prisoners in the province of Ontario, 2.1% and 1.8% of men and women were HIV positive, respectively [46].

In a study including 3930 prisoners in England and Wales, 14 prisoners (0.4%) were seropositive [47]. In two studies conducted in France, HIV prevalence was 6% among 391 inmates in Marseille and 2% among 2154 inmates of 27 prisons [48, 49]. The prevalence rate in some prisons in Italy was 7.5%, in a study including eight prisons in this country, it was higher in comparison to other studies we reviewed in this same region [50].

### 3.6 Latin America and the Caribbean

HIV epidemic in Latin America has remained stable for a number of years [51]. Although HIV prevalence in Latin American countries is relatively low, the number of infected people is still substantial. The majority of people living with HIV in Latin America (75%) are in four countries, including Brazil, Colombia, Mexico and Venezuela [21]. The prevalence of HIV among Brazilian prisoners is estimated about 3.2% according to results of one study performed in Manhuaçu, Minas Gerais [4]. Another study on 12 Brazilian prisons revealed that 1.6% of those prisoners were HIV positive [52]. Two studies in Mexico City included 17,084 and 4,268 prisoners found that the prevalence of HIV was 0.7% and 0.6%, respectively [15, 16].

## 4 CORRELATIONS OF HIV TRANSMISSION IN PRISONS: WHICH ARE THE RISK FACTORS

### 4.1 Sub-Saharan Africa

Based on one study in Zambia, demographic characteristics such as age, marital status, the numbers of irregular lifetime partners or pick-ups have been considered as correlated factors [21]. The most prevalent risk factors among prisoners in Ghana were illegal drug use, especially marijuana (83.2%), same sex practices among men (30.8%) and women (22.7%) [23]. History of sexually transmitted infections (STIs) was the only risk factor among 34 prisons in Uganda [24]. Clearly, some of these behaviors do not transmit the virus *per se*, but their association with the transmission is quite interesting.

### 4.2 Middle East and North Africa

History of opioid use in prison, advanced age (adjusted odds ratio: 2.79 for 25-34, 3.01 for 35-44, 4.62 for ≥ 45 yr.), injection drug use (IDU), tattooing, promiscuous heterosexuality and homosexuality, needle sharing, duration of imprisonment and drug use period had a significant correlation with HIV seropositivity in Iranian prisoners [27, 29-32].

### 4.3 Asia and Pacific

In a study in Pakistan, multiple contacts with Commercial Sex Workers (CSW), donating blood and injecting drug with reused syringes were important risk factors for HIV infection among prisoners [35]. History of injection, positive urine opiate test, history of attendance to drug detoxification centers and presence of tattoos on the body have been found associated with HIV prevalence in Thailand, by a multivariate analysis [37].

### 4.4 Western and Central Europe and North America

In Rhode Island, important risk factors were Black and Hispanic race/ethnicity, age >40 years and injection drug use [8]. In a prison in Scotland, all inmates living with HIV had shared injecting equipment inside the prison [42]. In a study in Ireland, 509 out of 1,178 respondents (43.2%) reported having ever injecting drugs and smoking heroin. In addition, of 492 injecting drug users, 347 (70.5%) reported sharing needles while they were in prison, and 28 (2.5%) out of 1,108 men reported having anal sex with another man before imprisonment; 17 individuals declared that they had never used condoms. Moreover, 20 participants among 1,079 men who answered the question reported having had anal sex with another man while they were in prison [6]. Injecting drug use, Black race, Hispanic ethnicity, psychiatric illness and a history of sexually transmitted disease showed positive correlations in a study in the USA [43].

In British Columbia, the prevalence rates were higher among women than men (3.3% v. 1.0%) and among the inmates who reported a history of injection drug use more than those who did not report it (2.4% v. 0.6%) [44]. In two studies conducted in Quebec, HIV correlations are mentioned as injecting drug use, with an HIV prevalence of 9% (11 out of 129) among male IDU and 16% in female IDU. Men who have sex with men (MSM) prior to incarceration showed 10% prevalence of HIV. Tattooing was not associated with HIV infection either in men or women, but injection drug use, needle sharing, tattooing and piercing with non-sterile equipment, unprotected sex with injection drug user, unprotected anal sex, and unprotected oral or anal or vaginal sex for money or drugs were other high-risk behaviors inside and outside the prisons [18, 45]. In addition, age ≥ 30 years and IDU are mentioned in a study in Ontario, Canada [46].

In a study in Italy, although HIV was strongly associated with IDU, after excluding IDUs and male homosexuals, the HIV prevalence remained relatively high (2.6%). HIV prevalence was higher in persons from Northern Italy and Sardinia. The age distribution was U-shaped for HIV infection, and the number of imprisonments per person was associated with HIV infection [50]. IDU with an HIV prevalence of 0.5% was a risk factor in England and Wales [47].

In Marseille, France, IDU alone was reported as a risk factor, with 21% of HIV prevalence [49], conversely, in another study in France, HIV prevalence increased with age (from 0% among 18-21 years old persons to 3.7% among the 41-50 years old group), and it varied according to continent of birth, being highest among individuals born in Sub-Saharan Africa (15.4%) [48].

### 4.5 Latin America and the Caribbean

Low socioeconomic and education status, the flexibility of moral values associated with the absence of motivation to improve self-destructive behavior, drug use, occasional multiple substances use and several sexual partners are the correlations described in a study in Brazil [4].

## 5 PERSISTENCE OF RISK BEHAVIORS

Many prisoners have been documented for having an infection with STIs and Hepatitis C at the time of entrance into the prison [53]. Women prisoners, due to take part in sex working, they might be at greater risk of STIs at the time of entrance into the prison [54]. Researchers also demonstrated that high risk sexual behaviors correlated with STIs and HIV transmission can occur during imprisonment. Since condom availability is rare in the prisons, even though the availability, it is uncommon to use a condom during sexual relationship [55].

The HIV prevalence and correlates among prisoners are summarized in Table **1**.

## 6 HIV PREVENTION AND TREATMENT IN PRISONS

Prison environment not only provides health services for HIV diagnosis among prisoners, but also is an opportunity for HIV prevention and treatment [56]. Numerous scientists suggested correctional facilities offer as an ideal opportunity for implementing HIV prevention interventions. On the contrary, when they are in the community, incarcerated individuals are logistically easier to reach with prevention and education programs; Supposedly, encountering fewer situations of risk (*e.g.*, sex relationship under the influence of drugs or alcohol, anonymous sex); they are sometimes re-evaluating their life choices; they have access to medical and mental health services for little or no cost; and they have fewer demands being made on their time [57]. Attempts to the HIV prevention in prisons have been slow in comparison to those in the surrounding communities. Nevertheless, HIV education in term of prevention intervention is widely done in prisons, although they were insufficient unless prevention programs like harm reduction are also provided [58]. In Maryland’s prevention case management program, individual and group counseling including education on HIV risks, condom and substance use all were provided to inmates nearing release from prison to reduce sexual risk behaviors. These interventions lead to change in condom attitudes, condom use self-efficacy and self-efficacy to reduce injection drug and other substance risk [59]. The Researchers in a study in an Indonesian prison conducted a prevention and treatment program, including HIV education, voluntary HIV testing, condom supply, prevention of rape, methadone maintenance therapy and antiretroviral therapy for positive cases. They reported some considerable results that showed no new HIV cases detected after implementation of the program also the mortality rate of HIV cases decreased [60].

The relatively slow development and implementation of HIV prevention programs in prison settings have occurred for several reasons, for instance, the duality and cultural divide between public health and corrections. Corrections focus on promoting the custody and security of inmates; if there are interests in providing medical services and inmate’s health, prevention programs are not in the priority list. Moreover, some of the prison officials prefer directly contradicting policies that prohibit anal sex, condom use, and injection drug use in prisons rather than performing HIV prevention programs like providing condoms and sterile syringes. However, this concern can be resolved by increasing knowledge about the necessity of HIV prevention in prisons. Additionally, the stigma associated with discussing HIV/AIDS causes inmates to fear of expressing an open interest in learning about HIV prevention strategies or requesting testing, that may cause negative people feel of them [57].

The United Nations Standard Minimum Rules for the Treatment of Prisoners (the Nelson Mandela Rules) believe that prisoners should receive the same treatment services as there is in the society [61]. Besides the fact that prisoners should receive Health Services for HIV infection during imprisonment, it is important to access HIV treatment and follow-up after discharge. A study in Russia showed that only 36% of prisoners who had received HIV treatment in prisons, continued anti-retroviral therapy (ART) after their imprisonment [62]. It happens especially for IDUs because their adherence to ART may not be their first priority after discharge.

One sample of improvement in Health Services for HIV positive individuals in Iranian prisons, called “Prison based Active Health Services Provision” (PAHSP), was designed to pursue infected prisoners from diagnosis to treatment and follow-up after discharge. For this purpose, prisoners are connected to triangular clinics to receive necessary services. These clinics are expected to seek infected prisoners actively, by other components of health care system in prisons and some volunteer prisoners. In a study which run to evaluate the efficacy of this model, the HIV positive prisoners who covered by PAHSP received significantly more Health Services than the control group [26].

As mentioned above, it is necessary for policy makers to consider prisons as appropriate places for prevention, diagnosis and treatment of HIV infection among prisoners and find a strategy to provide effective Health Services during and after imprisonment for prisoners as high-risk populations.

## CONCLUSION

Various methods and strategies have been used to collect data and analyze HIV prevalence among prisoners in different regions of the world. In general, data are difficult to compare. It seems that there is a significant difference in HIV prevalence among prisoners not only in different regions of the world, but also in various areas of a country. Certainly, all studies agree on the fact that HIV prevalence in prisons is significantly higher than in the surrounding communities [63-65]. Despite the fact that the high prevalence of HIV in prisoners represents a main sanitary problem almost in all societies, easier access to prisoners makes prisons worthy of effective interventions [64].

Although all efforts should be done to decrease the entry into prisons and lowering the prisons’ population [66], effective services and adequate therapeutic strategies must be pursued for prison inmates. As an example, adherence to ART in prisoners belonging to marginal population is better in prisons if compared to inmates who are released [64]. Thus, intramural educational programs are clearly needed to improve the adherence to therapy of these subjects outside the prison, and thus reduce the spread of the infection.

On the other hand, patterns of HIV transmission are various among different regions of the world and change over time within regions. However, injecting drug use is present almost everywhere. Previously effective evidence-based interventions have been introduced to prevent HIV transmission among IDUs. Eventually, applying evidence-based interventions like substitution of syringes can decrease HIV transmission among IDUs to zero or close to that [67]. It must be underlined that in this population HIV prevalence can be expanded by explosive speed without effective interventions. There are indeed evidence-based recommendations to curtail HIV transmission across prisons. Emphasizing the need of expanding substance maintenance treatment, providing low-risk injection and optimizing antiretroviral therapy regimens are crucial parts of these activities [68-76].

Finally, sexual transmission of HIV is also an important route of transmission in the most studies, but it seems that actions to prevent sexual transmission are often neglected. Therefore, improving condom promotion program is a necessity everywhere [63]. In addition, since tattooing is an important factor in predicting the risk of HIV transmission in some areas, also allowing safe tattoo in the prisons should be considered.

## Figures and Tables

**Table 1:** HIV prevalence and the correlates among prisoners in different regions of the world.

**Region**	**Country-City Name**	**Authors**	**Target Group**	**Prevalence**	**Correlations**
**Sub-Saharan Africa**	Ghana, Nsawam and Accra [23]	Adjei AA, *et al.*	281 inmates	19.2%	Used illicit drugs, especially marijuana (83.2%), practiced homosexuality (30.8%) and lesbianism (22.7%)
	Zambia [22]	Simooya, *et al.*	1596 inmates (46 women)	421 (27%)	Age, marital status, the number of irregular lifetime partners or pick-ups
	Uganda [24]	UNODC	459 prisoners from 34 prisons and 85 health workers	11%	History of STD
	Burkina Faso [14]	Diendéré EA. *et al.*	300 prisoners in Burkina Faso	5%	Razor blades (20%), toiletries blades (18.7%) and drug abuse (14.6%)
**Middle East and North Africa**	Iran [27]	Navadeh S, *et al.*	5,530 prisoners from 27 prisons	2.1%	History of drug injection, tattooing and age over 30 years
	Iran, Bandar Abbas and Roodan [29]	Davoodian P, *et al.*	252 IDU prisoners	15.1%	Duration of imprisonment and drug use
	Iran, Gorgan [30]	Khodabakhshi B, *et al.*	121 IDU prisoners	5.8%	IDU, tattooing and shared syringes, promiscuous heterosexuality and homosexuality
	Iran, Isfahan [31]	Dibaj R, *et al.*	970 IDU prisoners	6.4%	Needle sharing
	Iran, Tehran [32]	Kheirandish P, *et al.*	459 male IDU arrested by police	24.4%	History of using opioid in jail and older age
**Asia and Pacific**	Australia, Victoria [11]	Crofts N, *et al.*	3627 prison entrants (3429 male and 198 female)	0.47%	HIV prevalence was higher among IV drug users.
	India [36]	Dolan K and Larney S	Indian prisoners	1.7%	Sex between inmates was reported common.
	Pakistan [34]	Salman S, *et al.*	4897 prisoners	1%	-
	Pakistan, Sindh [35]	Baqi, S, *et al.*	3525 prisoners	1 of 3441 male and 1 of 84 female	Multiple contacts with commercial sex workers, donating blood, injecting drug with re-used syringes
	Thailand, Bangkok [37]	Thaisri H, *et al.*	689 male inmates	HIV incidence: 4.18 per 100 p/y among all inmates, and 11.10 per 100 p/y among the IDUs	History of injection, positive urine opiate test, history of attendance to drug withdrawal clinics and the presence of tattoos on the body
**Eastern Europe and Central Asia**	Greece, Patra [9]	Malliori M, *et al.*	544 drug users imprisoned for drug related offences	0.19%	Injecting drug use and needle sharing
**Western and Central Europe and North America**	Canada, British Columbia [44]	Diane AR	2482 prisoners	1.1%	Rates of prevalence were higher among women than men (3.3% vs. 1.0%) and among the inmates who reported a history of injection drug use than those who did not report it (2.4% vs. 0.6%).
	Canada, Ontario [46]	Calzavara L, *et al.*	1877 prisoners	2.1% in men and 1.8% in women	Older offenders (≥ 30 years) and injection drug users
	Canada, Quebec [18]	Dufour A, *et al.*	618 inmates	2% in men and 8% in women	All HIV-infected men with history of injecting drugs showed an HIV prevalence of 9%. HIV prevalence amongst men reporting sexual intercourse with other men prior to incarceration was 10%. HIV prevalence was 16% in female IDU.
	Canada, Quebec [45]	Poulin C, *et al.*	1607 inmates (1357 men and 250 women)	2.3% among male and 8.8% among female	Injection drug use, needle sharing, tattooing and piercing with non-sterile equipment, unprotected sex with injection drug user, unprotected anal sex and unprotected oral, anal or vaginal sex for money or drugs
	England and Wales [47]	Weild AR, *et al.*	3930 prisoners	0.4%	-
	France [48]	Semaille C, *et al.*	2,154 inmates from 27 prisons	2.0% in men and 2.0% in women	Raised HIV prevalence was occurred from 0% among young 18–21 to 3.7% of the 41–50 years old.
	France, Marseille [49]	Rotily M, *et al.*	391 prisoners	6%	The rate of prevalence was 21% among IDUs.
	Ireland [6]	Allwright S, *et al.*	1205 prisoners (57 women)	2%	Among 1178 respondents, 43% reported using injecting drugs and smoking heroin. Moreover, 71% out of 492 injecting drug users, shared and exchanged needles in prison. Three percent out of 1108 men reported having anal sex with another man before committal, while 17 persons stated that never have before used condoms, and 20 of the 1079 men who answered the question they have had anal intercourse with another man while they were in prison.
	Italy [50]	Babudieri S, *et al.*	973 inmates from 8 prisons	7.5%	IDU and number of imprisonments. The relative age effect was U-shaped. HIV prevalence was higher in persons from Northern Italy and Sardinia than other parts.
	Rhode Island [8]	Macalino GE, *et al.*	4269 male inmates	1.2%	Black and Hispanic ethnicity, older than 40 years of age and injecting drug use
	Scotland, HM Prison Glenochil [42]	Taylor A, *et al.*	378 inmates	7%	All infected inmates had shared injecting tools within the prison.
	USA [43]	Altice FL, *et al.*	975 newly sentenced prisoners	6.1%	Injection drug use, black race, Hispanic ethnicity, psychiatric illness and a history of having a sexually transmitted disease
**Latin America and the** **Caribbean**	Brazil, Manhuaçu, Minas Gerais [4]	Corrêa Catalan-Soares B, *et al.*	63 male prisoners	3.2%	Low socioeconomic and education status, the flexibility of their moral values associated with the absence of motivation to improve self-destructive behavior, drug use, occasional multiple substances use and several sexual partners
	Brazil [52]	Sgarbi RVE., *et al.*	3362 inmates from 12 prisons	1.6%	Homosexuality, history of STIs, positive syphilis serology and history of mental illness
	Mexico, Mexico City [15]	Bautista-Arredondo S., *et al.*	15,354 men and 1,730 women	0.7% of men and 0.8% of women	Smoking, drug use, physical, sexual, or emotional violence during their lifetime, sex work and low rates of condom use.
	Mexico [16]	Belaunzaran-Zamudio PF., *et al.*	4,268 inmates	0.6%	STIs, exchanging materials for tattoo and injecting drugs, using unprotected heterosexual and homosexual intercourse.
	Brazil, Sao Paulo [17]	Maerrawi I., *et al.*	514 individuals	1.8%	Injectable drug use, age over 30 years, cocaine use and crack-cocaine use.
